# miR-520h Stimulates Drug Resistance to Paclitaxel by Targeting the OTUD3-PTEN Axis in Breast Cancer

**DOI:** 10.1155/2020/9512793

**Published:** 2020-05-09

**Authors:** Wenwen Geng, Haiyun Song, Qianqian Zhao, Ke Dong, Qian Pu, Haidong Gao, Yanrong Lv

**Affiliations:** ^1^Department of Breast Surgery, Qilu Hospital (Qingdao) of Shandong University, Qingdao, Shandong 266000, China; ^2^Department of Pathology, Qilu Hospital (Qingdao) of Shandong University, Qingdao 266000, China; ^3^Department of Breast Surgery, Qilu Hospital of Shandong University, Jinan, Shandong 250012, China

## Abstract

MicroRNAs (miRNAs) have been identified as negative posttranscriptional regulators of target genes and are involved directly in the pathological processes of tumors, including drug resistance. However, the exact function of miR-520h in breast cancer remains poorly understood. The aim of this study was to investigate the molecular mechanisms of miR-520h in paclitaxel resistance in the MCF-7 breast cancer cell line. Ectopic expression of miR-520h could promote the proliferation of breast cancer cells and inhibit paclitaxel-induced cell apoptosis. Inhibiting the expression of miR-520h could enhance the sensitivity to paclitaxel in paclitaxel-resistant MCF-7/Taxol cells. Furthermore, luciferase reporter assays showed that OTUD3 was a direct target of miR-520h. OTUD3 plays a necessary role in the paclitaxel resistance effect of miR-520h, and cotreatment with a miR-520h inhibitor and OTUD3 overexpression significantly enhanced MCF-7 cell sensitivity to paclitaxel. Moreover, miR-520h substantially inhibited the protein expression of PTEN via OTUD3 and subsequently affected downstream p-AKT pathway activity. In a clinical study, we also found that high miR-520h expression was associated with more aggressive pathological characteristic and poor prognosis. Therefore, our findings showed that miR-520h targeted the OTUD3-PTEN axis to drive paclitaxel resistance, and this miR might be an important potential target for breast cancer treatment.

## 1. Introduction

Breast cancer is one of the most common invasive malignancies in women worldwide [1]. Despite the development of various breast cancer treatment strategies, this disease still ranks second among the most common causes of cancer death in women [2]. Chemotherapy is widely used for treating breast cancer, either before or after surgery [3]. However, drug resistance strongly limits its efficacy and can cause systemic treatment failure [4]. Therefore, elucidation of the chemoresistance mechanisms is urgently needed to overcome these limitations and improve better breast cancer patient survival.

MicroRNAs (miRNAs) are endogenous small noncoding RNAs (~18-22 nucleotides) [5]. By binding to the 3′-untranslated region (3′-UTR) of target mRNAs, miRNAs can induce mRNA degradation or translational inhibition of functional proteins [6]. Over the past few years, miRNAs have been shown to be involved in tumorigenesis, metastasis, and tumor response to treatment [7–9]. In particular, many miRNAs have been reported to play critical roles in breast cancer progression [10, 11]. Previous studies showed that miR-520h acts as an oncogenic miRNA, and its downregulation by E1A is critical for E1A-mediated tumor and invasion suppression [12].

We previously reported that OTUD3, a deubiquitinase, could stabilize PTEN by depolyubiquitination [13]. Depletion of OTUD3 could activate the p-AKT signaling pathway, inducing cellular transformation and cancer metastasis. Furthermore, the expression levels of OTUD3 and PTEN have been correlated with human breast cancer progression. Our findings demonstrated that OTUD3 is an essential regulator of PTEN and that the OTUD3-PTEN signaling axis plays a critical role in tumor suppression. However, the role of the OTUD3-PTEN signaling axis in drug resistance and its relationship with miRNAs are unclear.

## 2. Results

### 2.1. miR-520h Stimulates Drug Resistance to Paclitaxel in Breast Cancer Cells

**Figure 1:**
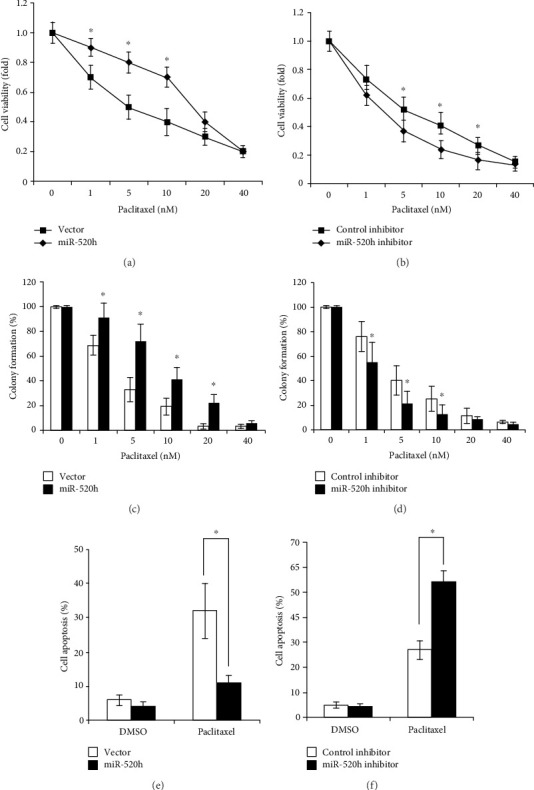
miR-520h expression promotes drug resistance in human breast cancer cells. (a) Viability of MCF-7/Vec and MCF-7/miR-520h cells after exposure to paclitaxel. (b) Viability of MCF-7 cells with miR-520h inhibition after treatment with increasing concentrations of paclitaxel. (c) The clonogenic ability of MCF-7/Vec and MCF-7/miR-520h cells after treatment with various concentrations of paclitaxel. (d) The clonogenic ability of MCF-7 cells with miR-520h inhibition after treatment with various concentrations of paclitaxel. (e) MCF-7 cells were transfected with vector or miR-520h and treated with 5 nM of paclitaxel for 24 h and then the apoptosis assayed by flow cytometry analysis. (f) MCF-7 cells were transfected with control or miR-520h inhibitor and treated with 5 nM of paclitaxel for 24 h and then the apoptosis assayed by flow cytometry analysis. Results were collected from three independent experiments, with triplicate repeats for each experiment. ^∗^*P* < 0.05, ^∗∗^*P* < 0.01.

**Figure 2:**
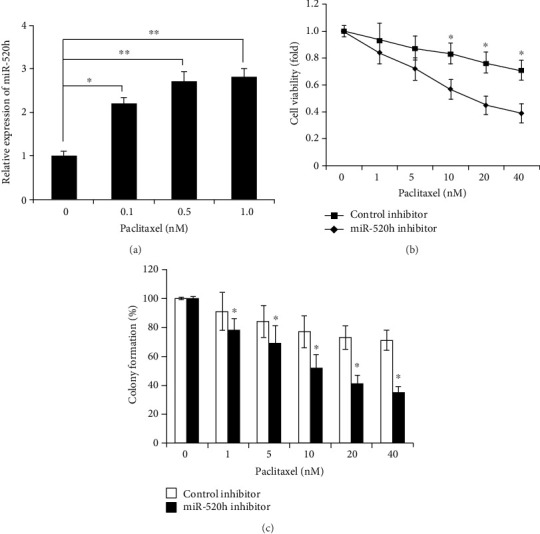
Inhibition of miR-520h expression can reverse the cell resistance to paclitaxel in drug-resistant breast cancer cells MCF-7/Taxol. (a) The miR-520h expression of MCF-7 cells treated with various concentrations of paclitaxel. (b) The viability of MCF-7/Taxol cells after inhibition of miR-520h expression. (c) The colony formation ability of MCF-7/Taxol cells after inhibition of miR-520h expression. Results were collected from three independent experiments, with triplicate repeats for each experiment. ^∗^*P* < 0.05.

RT-qPCR was conducted to examine miR-520h levels in MCF-7 cells after transfection. A significant increase in miR-520h levels was observed following transfection with miR-520h, while treatment with a miR-520h inhibitor led to a significant decrease in the miR-520h level in MCF-7 cells. To investigate the biological role of miR-520h in paclitaxel resistance in breast cancer, an MTS assay was performed to examine cell viability in each group. Overexpression of miR-520h significantly promoted MCF-7 cell viability upon paclitaxel treatment (*P* < 0.05, Figure 1(a)). To investigate the necessity of endogenous miR-520h for paclitaxel resistance, we used a specific inhibitor targeting miR-520h to decrease the level of endogenous miR-520h. The results showed that MCF-7 cells with miR-520h inhibition were more sensitive to paclitaxel treatment (*P* < 0.05, Figure 1(b)). Furthermore, a colony formation assay was conducted to detect the effects of miR-520h on MCF-7 cell growth. Overexpression of miR-520h significantly improved the clonogenic ability of MCF-7 cells (*P* < 0.05, Figure 1(c)). In contrast, the clonogenic ability was significantly lower in the breast cancer cells treated with the miR-520h inhibitor than in the control cells (*P* < 0.05, Figure 1(d)). Overexpression of miR-520h could significantly attenuate the apoptosis of paclitaxel-induced breast cancer cells (*P* < 0.05, Figure 1(e)). However, after endogenous miR-520h inhibition with a specific inhibitor, the MCF-7 cells showed increased sensitivity to paclitaxel treatment (*P* < 0.05, Figure 1(f)). In addition, the response of miR-520h to different concentrations of paclitaxel in the MCF-7 breast cancer cells was analyzed. We found that the expression of miR-520h was significantly upregulated with increasing concentrations of paclitaxel (*P* < 0.01, Figure 2(a)). Therefore, we further examined the role of miR-520h in the paclitaxel-resistant breast cancer cell line MCF-7/Taxol. The results showed that after inhibition of miR-520h by an inhibitor in drug-resistant breast cancer cells, paclitaxel was effective in these cells. The inhibition to cell proliferation and colony formation induced by paclitaxel was significantly enhanced (*P* < 0.05, Figures 2(b) and 2(c)), which indicated that inhibition of miR-520h expression can reverse the cell resistance to paclitaxel in drug-resistant breast cancer cells. Therefore, our results showed that miR-520h could promote the survival of breast cancer cells and attenuate the apoptosis upon paclitaxel treatment. Thus, this miR plays a positive role in drug resistance to paclitaxel in breast cancer.

### 2.2. miR-520h Targets OTUD3 and Suppresses OTUD3-PTEN Expression

**Figure 3:**
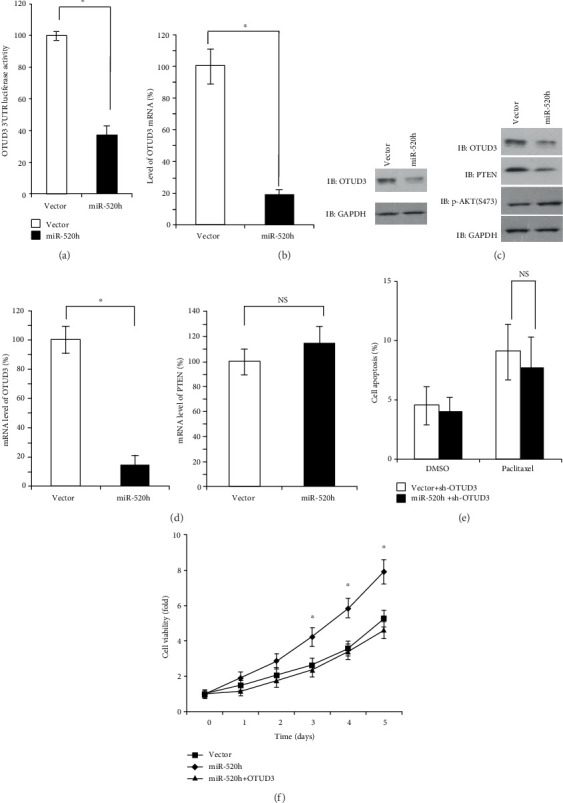
OTUD3 is targeted by miR-520h and associated with the miR-520h-mediated drug resistance. (a) Luciferase activity of OTUD3 3′UTR in MCF-7 cells transfected with miRNA-520h. (b) Effects of miR-520h overexpression on the expression of OTUD3 mRNA and protein. (c) Expression of OTUD3, PTEN, and p-AKT in MCF-7 breast cancer cells was detected by Western blot. (d) The OTUD3 and PTEN levels were determined by RT-PCR analysis after miR-520h overexpression. (e) The apoptosis of MCF-7/sh-OTUD3/Vec, MCF-7/sh-OTUD3/miR-520h treated with 5 nM of paclitaxel for 24 h and then assayed by flow cytometry analysis. (f) Overexpression of OTUD3 significantly inhibits miR-520h-induced MCF-7 cell viability compared to the control group. Results were collected from three independent experiments, with triplicate repeats for each experiment. ^∗^*P* < 0.05, ^∗∗^*P* < 0.01.

We used several online databases, including TargetScan, miRBase, and miRGen, for miRNA target gene prediction. The results indicated that OTUD3 contains miR-520h binding sites. A luciferase reporter assay demonstrated that the relative luciferase activity of the OTUD3 3′-UTR was substantially reduced by miR-520h transfection (*P* < 0.05, Figure 3(a)). Western blotting was performed to examine the protein levels of OTUD3 in miR-520h-overexpressing MCF-7 cells, and the results showed that miR-520h expression significantly decreased the endogenous OTUD3 protein levels. Likewise, miR-520h expression reduced the OTUD3 mRNA levels according to qRT-PCR, suggesting that miR-520h targets OTUD3 mRNA for degradation (*P* < 0.05, Figure 3(b)). Furthermore, we noted that PTEN protein expression was downregulated and that p-AKT protein was upregulated after miR-520h overexpression (Figure 3(c)). PTEN is a tumor suppressor with a positive relationship with OTUD3 due to depolyubiquitylation, and it is regulated at the posttranscriptional level. However, as the mRNA and protein levels of OTUD3 were both suppressed by miR-520h overexpression, PTEN mRNA expression was not significantly affected (NS, Figure 3(d)), which indicated that PTEN expression might be regulated by OTUD3 rather than by miR-520h. Therefore, miR-520h might directly mediate the expression of OTUD3 at the transcriptional level in breast cancer cells.

### 2.3. OTUD3 Is Involved in miR-520h-Mediated Paclitaxel Resistance

To determine whether OTUD3 is essential for miR-520h-mediated paclitaxel resistance, we adopted OTUD3 shRNA to mimic miR-520h-mediated OTUD3 downregulation. Under these conditions, the drug resistance effects of miR-520h were significantly inhibited by the deletion of OTUD3, and paclitaxel-induced apoptosis in MCF-7 cells was not significantly different between the miR-520h overexpression and negative control groups (NS, Figure 3(e)). Furthermore, MCF-7 cells were transfected with OTUD3 to increase OTUD3 expression, and this reexpression of OTUD3 abrogated MCF-7/miR-520h cell resistance to paclitaxel-induced apoptosis. MTS assays indicated that OTUD3 overexpression significantly inhibited the miR-520h-induced MCF-7 cell viability in response to paclitaxel treatment (*P* < 0.05, Figure 3(e)). Based on the results of the present study, OTUD3 might play a crucial role in miR-520h-mediated paclitaxel resistance.

### 2.4. miR-520h Regulates OTUD3-PTEN and Breast Cancer Progression

**Figure 4:**
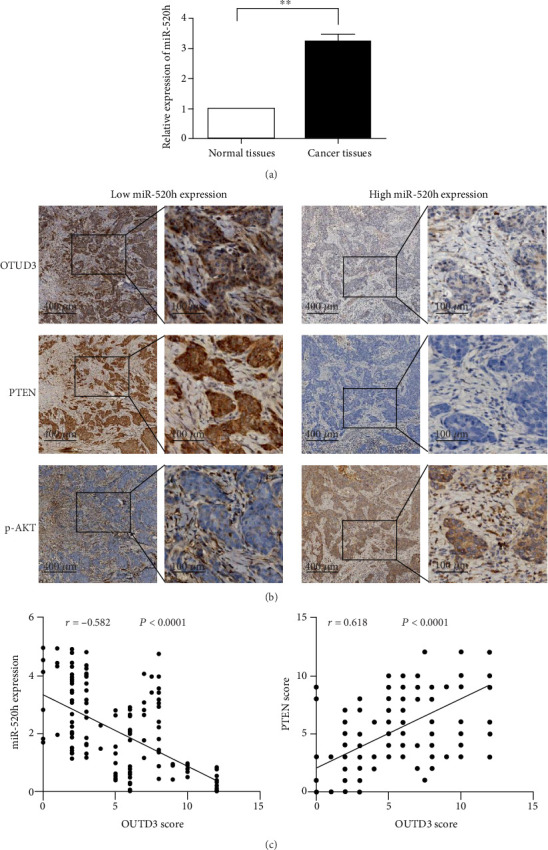
miR-520h, OTUD3, PTEN, and p-AKT expression in breast cancer tissues. (a) Relative expression of miR-520h in normal breast vs. breast cancer tissues. (b) The expression of OTUD3, PTEN, and p-AKT in breast cancer tissues of different miR-520h levels. (c) Correlation analysis of the expression of OTUD3 with miR-520h and PTEN. ^∗^*P* < 0.05.

**Figure 5:**
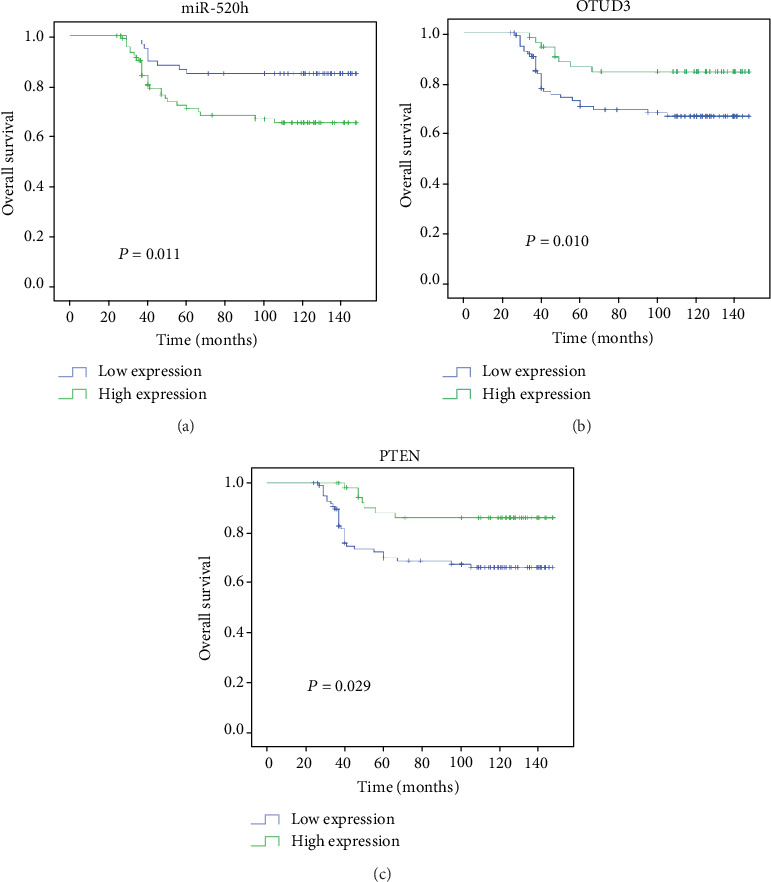
Kaplan-Meier plot showing a significant association of miR-520h, OTUD3, and PTEN expression with overall survival for breast cancer patients. (a) High expression level of miR-520h associates with poor clinical outcome in the 156 patients with breast cancer (*P* = 0.011). (b, c) Conversely, OTUD3 and PTEN expression predicts better survival (*P* = 0.010, *P* = 0.029). (c) The association of PTEN expression with overall survival for breast cancer patients.

To determine whether miR-520h expression and the OTUD3-PTEN axis are associated with the clinicopathological characteristics of breast cancer patients, 156 pairs of tumor tissues and adjacent normal tissues were analyzed by RT-PCR and immunohistochemistry (IHC). miR-520h expression levels were higher in the tumor tissues (96/156) than in the normal breast tissues as assessed by RT-PCR analysis (*P* < 0.01, Figure 4(a)). According to the IHC analysis, the OTUD3 and PTEN expression levels were inversely correlated with p-AKT. OTUD3 and PTEN were highly expressed in the breast tissues with low miR-520h expression, while p-AKT was expressed at low levels. However, in the tumor tissues with high miR-520h expression, OTUD3 and PTEN were expressed at low levels, and p-AKT was highly expressed (Figure 4(b)). Correlation analysis showed that the expression of miR-520h was negatively correlated with the expression of OTUD3 (*r* = −0.582, *P* < 0.001), and the expression of PTEN was positively correlated with the expression of OTUD3 (*r* = 0.618, *P* < 0.001) (Figure 4(c)). Further research showed that significant correlations were observed between miR-520h/OTUD3/PTEN expression and histological grade, tumor size, and lymph node status (Table 1). We found that patients with high levels of miR-520h exhibited advanced tumor size (*P* = 0.041), high histological grade (*P* < 0.27), and increased lymph node metastasis (*P* = 0.030). In contrast, OTUD3 expression was negatively associated with high histological grade (*P* = 0.009), T2-T3 (>2 cm) tumors (*P* = 0.007), and lymph node metastasis (*P* = 0.025). However, PTEN expression was only negatively associated with T2-T3 (>2 cm) tumors (*P* = 0.001). Associations between miR-520h/OTUD3/PTEN expression and patient age, estrogen receptor (ER) status, progesterone receptor (PR), and HER2 status were not statistically significant. A log-rank analysis indicated that high miR-520h expression in the tumor tissues predicted poor survival in breast cancer patients (*P* = 0.011, Figure 5(a)), whereas patients with high expression levels of OTUD3 (*P* = 0.010, Figure 5(b)) and PTEN (*P* = 0.029, Figure 5(c)) had better survival rates than those with low expression levels.

## 3. Discussion

Despite advanced therapy strategies and improved early surveillance of breast cancer, drug resistance remains an important reason for systematic treatment failure [4]. The mechanisms responsible for drug resistance have been elucidated in multiple studies, and increasing evidence has demonstrated that miRNAs contribute to therapeutic resistance in cancers [14–16]. It has been reported that miR-100, miR-101, and miR-26a are associated with paclitaxel resistance in breast cancer cells [17–19]. Furthermore, miR-520h was overexpressed in epithelial ovarian cancer (EOC) tissues, and high miR-520h expression predicts poor prognosis for patients, especially those with disease progression. Moreover, miR-520h could promote cell proliferation and suppress apoptosis in EOC cells [20]. Other research showed that miR-520h could inhibit cancer progression and overcome drug resistance in some cancers [21–23]. miR-520h could play a tumor suppressive role in gastric tumors by targeting HDAC1. Another study showed that miR-520h acts as a tumor suppressor in PANC-1 pancreatic cancer cell by regulating cell proliferation, cell cycle progression, apoptosis, migration, and invasion. Recently, Su showed that miR-520h could enhance the chemoresistance of breast cancer cells to paclitaxel by targeting DAPK2 [24] and that miR-520h expression might be an indicator of poor prognosis in breast cancer patients. However, a single target gene is often controlled by multiple miRNAs, and one miRNA might target many genes.

In this study, we focused on the role of miR-520h in paclitaxel resistance in breast cancer cells and impact on the prognosis of breast cancer patients. According to MTS and flow cytometric assays, our results demonstrated that miR-520h overexpression promoted cell growth but inhibited cell apoptosis induced by paclitaxel. In contrast, downregulation of miR-520h enhanced the paclitaxel sensitivity in both the MCF-7 breast cancer cells and the paclitaxel-resistant breast cancer cell line MCF-7/Taxol. Thus, our results showed that miR-520h played a crucial role in the paclitaxel resistance of breast cancer. To further explore the mechanism by which miR-520h regulates paclitaxel resistance, we predicted its target genes through bioinformatics analysis. The results demonstrated that miR-520h specifically targeted the OTUD3 3′UTR and suppressed the protein expression of OTUD3 in breast cancer cells. However, ectopic expression of OTUD3 could reverse the drug resistance induced by miR-520h. Thus, our results indicated that miR-520h could regulate drug resistance to paclitaxel by targeting OTUD3 in breast cancer cells. OTUD3 regulation by miRNAs has never been illustrated in breast cancer. This study identifies for the first time that miR-520h can suppress OTUD3 expression via epigenetic regulation.

OTUD3 is one member of the deubiquitinase (DUB) family, and researchers have shown that the ubiquitination of many proteins can be reversed by the DUBs. In our previous study, we showed that OTUD3 exhibited a tumor suppressor role and acted as a specific cytoplasmic deubiquitinase for PTEN in breast cancer. PTEN is considered one of the most commonly mutated genes in human cancers, because even slight changes in PTEN might cause tumor initiation and progression, especially for the development of breast cancer. PTEN is a key regulator of the PI3K/p-AKT/mTOR pathway and acts as a PI3K inhibitor [25]. Based on mediation of the signals of multiple growth factors, the PI3K/p-AKT/mTOR pathway has been shown to play a pivotal role in breast cancer metastasis and drug resistance [26]. Reduction or loss of PTEN is involved in breast cancer progression and drug resistance [27]. Our results showed that miR-520h strongly inhibited the expression of PTEN via OTUD3 and subsequently affected downstream p-AKT pathway activity in vitro. Activation of the AKT pathway could enhance the proliferation and invasiveness of breast cells, which might explain why high miR-520h expression was associated with aggressive clinicopathologic characteristics in the clinical study. Furthermore, the results showed that miR-520h was highly expressed in the breast cancer tissues compared with the adjacent tissues, which suggested that the expression of miR-520h might be related to the development of breast cancer. In contrast, the expression levels of OUTD3 and PTEN were relatively low in the breast cancer tissues. Further analysis showed that the expression of OTUD3 was negatively correlated with miR-520h but positively correlated with PTEN. Prognostic analysis showed that high expression of miR-520h is an indicator of poor prognosis for breast cancer patients, and breast cancer patients with high expression of OTUD3 and PTEN had a better prognosis than those with low expression. Therefore, our research indicates that miR-520h may be one of the prognostic indicators for breast cancer patients and further confirmed the miR-520h-OTUD3-PTEN regulatory axis in breast cancer. In this study, we demonstrated that the mechanism through which miR-520h induces drug resistance involves the OTUD3-PTEN axis. However, the roles of miR-520h in breast cancer patients with disease progression after paclitaxel treatment have not been explored, so further studies are needed to verify the findings of this study.

Our findings suggested that miR-520h increased breast cancer cell resistance to paclitaxel mainly through inhibiting OTUD3 expression, which subsequently attenuated PTEN stability and activated the AKT signaling pathway. Importantly, identification of the mechanism of drug resistance regulated by miRNAs will be helpful in finding new targets for the treatment of breast cancer.

## 4. Materials and Methods

### 4.1. Cell Culture and Transfection

MCF-7 cells were purchased from ATCC (Manassas, VA, USA), and MCF-7/Taxol cells were purchased from GeneChem (Shanghai, China). The cells were routinely maintained in DMEM supplemented with 10% fetal bovine serum, 50 U/ml penicillin, and 0.1 mg/ml streptomycin at 37°C in a humidified 5% CO_2_ atmosphere. Cells were transfected with various plasmids using Lipofectamine 2000 (Invitrogen, Carlsbad, CA, USA) according to the manufacturer's protocol.

### 4.2. Patients and Immunohistochemistry

A total of 156 patients who underwent surgical treatment at the Breast Department of Qilu Hospital of Shandong University from January 2004 to May 2016 were recruited. All of the patients were female, and their ages ranged between 24 and 76 years, with an average age of 45.2 ± 9.2 years. The exclusion criteria were as follows: age younger than 18 years, incomplete clinical follow-up data, presence of other malignant tumors, and previous preoperative chemotherapy or radiotherapy. All included patients underwent surgery: either modified radical mastectomy (117, 75%) or breast-conserving therapy (39, 25%). After surgery, the patients were treated with chemotherapy or radiotherapy. The indications for postoperative chemotherapy, radiotherapy, or chemoradiotherapy were patients with various pathological features. Positive hormone receptor patients received adjuvant endocrine therapy for 5 years, and positive human epidermal growth factor receptor 2 (HER2) patients received trastuzumab targeted therapy for 1 year. After the treatment finished, the patients were regularly followed up with clinical examination and imaging. The patients were scheduled for clinical visits every 4-6 months during the first and second years; every 6 months during the third, fourth, and fifth years; and annually thereafter. A total of 156 paired breast cancer tumor tissues and matched adjacent normal tissues were collected from the patients. Immunohistochemistry assays were performed as previously described [13].

### 4.3. RNA Extraction and miRNA Analysis

Total cell RNA was prepared using a TRIzol reagent (Invitrogen) and from paraffinized tissues using a miRNeasy FFPE Kit (Qiagen, Hilden, Germany) according to the manufacturer's instructions. Reverse transcription reactions were performed using 1 *μ*g of total RNA and a High-Capacity cDNA Reverse Transcription Kit (Applied Biosystems, Foster City, CA, USA) or a Mir-X miRNA First-Strand Synthesis Kit (Takara, Dalian, China). Real-time quantitative RT-PCR was performed using a SYBR Premix Kit (Takara). Human glyceraldehyde-3-phosphate dehydrogenase (GAPDH) or U6 was used as an internal control for mRNA or miRNA quantification. The following primers were used: OTUD3: forward 5′-TAAAGCAGCGGGAAGATTTTGA-3′ and reverse 5′-TGCGATGTGTAACTCCCTCAC-3′; PTEN: forward 5′-TGGATTCGACTTAGACTTGACCT-3′ and reverse 5′-GGTGGGTTATGGTCTTCAAAAGG-3′; and GAPDH: forward 5′-GGAGCGAGATCCCTCCAAAAT-3′ and reverse 5′-GGCTGTTGTCATACTTCTCATGG-3′. The primers for miR-520h were purchased from Takara.

### 4.4. miRNA Inhibition and Overexpression

The miRNA expression vector or control vector was transfected into MCF-7 cells using Lipofectamine 2000 (Invitrogen) according to the manufacturer's instructions. For the miRNA inhibition studies, MCF-7 cells were transfected with miR-520h inhibitor (GeneChem, Shanghai, China) based on the manufacturer's instructions. Twenty-four hours after transfection, the cells were used for MTS, colony formation, and flow cytometry assays.

### 4.5. Lentivirus Infection

Recombinant lentiviruses were produced by cotransfecting a mixture of the expression plasmid GV115 (GeneChem, Shanghai, China). Virus production was carried out using HEK293T cells. Briefly, cells were transfected with lentiviral DNA constructs combined with the lentiviral packaging plasmids pHelper 1.0 and pHelper 2.0 at a ratio of 4 : 3 : 2. The viral supernatants were harvested 48 h after transfection, filtered using a 0.45 *μ*m pore filter, and then used for infection. To establish stable cell lines, MCF-7 cells were infected with either GFP-tagged OTUD3 knockdown or control lentivirus and selected with puromycin to obtain single-cell clones. The single-cell clones were cultured for 2 weeks and amplified for further experiments.

### 4.6. Cell Viability by MTS Assay

Cells were plated in 96-well plates (100 *μ*l of cell suspension, 1 × 10^4^ cells/ml) and subsequently incubated with the indicated drug concentrations. Twenty-four hours later, 0.05 mg/ml MTS reagent (Promega, Madison, WI, USA) was added to each well and incubated at 37°C for 4 h, followed by absorbance measurement at 490 nm. The values were standardized to wells containing media alone.

### 4.7. Cell Apoptosis by Flow Cytometry Analysis

Cells were treated with the indicated paclitaxel concentrations and incubated for 24 h. The apoptotic cells were then washed with PBS and stained with fluorescein isothiocyanate-Annexin V and propidium iodide according to the manufacturer's protocol (Beijing Biosea Biotechnology Annexin V Kit). Apoptotic cells (Annexin V-positive) were then determined by flow cytometry.

### 4.8. Colony Formation Assay

Cells were treated with various concentrations of paclitaxel for 24 h, and the medium was then replaced with fresh. A total of 5 × 10^2^ cells were seeded in six-well tissue culture dishes. After 2 weeks of culture, the colonies were stained with a crystal violet solution. Images were taken of the stained plates, and the colonies containing more than 50 cells were counted. Each treatment was performed in triplicate.

### 4.9. Western Blot Analysis

Cell lysates were created using radioimmunoprecipitation assay (RIPA) buffer (150 mM NaCl, 1% NP-40, 0.5% deoxycholic acid, 0.1% sodium dodecyl sulfate (SDS), 50 mM Tris-HCl pH 7.6) containing a protease/phosphatase inhibitor (Cell Signaling Technology, Danvers, MA, USA). Protein concentrations were quantified using a bicinchoninic acid (BCA) protein assay (Pierce Biotechnology, Rockford, Illinois, USA). Approximately 30 *μ*g of total protein from each sample was boiled for 10 min, separated by SDS-PAGE, and then transferred to PVDF membranes (Millipore, Billerica, MA, USA). After blocking with 5% nonfat milk, the membranes were incubated overnight at 4°C with primary antibodies against anti-PTEN (Abcam, Cambridge, MA, USA, 1 : 500), anti-OTUD3 (Abcam, 1 : 100), anti-pSer473-AKT (Cell Signaling Technology, 1 : 1,000), and GAPDH (Abgent, San Diego, CA, USA; 1 : 1,000). After washing and incubating with secondary antibodies (Sigma Aldrich, Darmstadt, Germany, 1 : 10000), the blots were visualized with an ECL reagent (Millipore).

### 4.10. Construction of Luciferase Reporters and Reporter Assay

After amplification, the miR-520h binding site OTUD3 3′-UTR cDNA fragment was cloned into the pGL3 luciferase vector (Promega). MCF-7 cells were transfected with miR-520h mimics together with the pGL3/vector. These cells were cultured for 24 h, and the luciferase activity was determined and recorded using a luminometer (Promega).

### 4.11. Statistical Analysis

All results are shown as the mean ± SD of multiple independent experiments. Student's *t*-test (for two group comparisons) or one-way ANOVA (for more than two group comparisons) was used for statistical analyses. A correlation analysis was performed with Pearson's correlation coefficient analysis. Statistical analyses of clinicopathological data were performed using Pearson's *χ*^2^ test. Survival curves were derived from Kaplan-Meier estimates, and we compared the curves using log-rank tests. All statistical analyses were performed with GraphPad Prism 8 and SPSS 19.0 software. All statistical tests were two-sided, and *P* values *<* 0.05 were considered statistically significant.

## Figures and Tables

**Table 1:** Relationship between miR-520h, OTUD3, and PTEN expression and the clinicopathologic characteristics in breast cancer.

Clinicopathologic characteristics	miR-520h		*P*	OTUD3		*P*	PTEN		*P*
	High	Low		High	Low		High	Low	
Age			0.079			0.308			0.745
≤50	49	22		22	49		26	45	
>50	47	38		33	52		29	56	
Tumor size			0.041			0.007			0.001
≤2 cm	48	40		39	49		41	47	
>2 cm	48	20		16	52		14	54	
Histological grade			0.027			0.009			0.300
I	32	28		30	30		25	35	
II	42	13		15	39		15	39	
III	23	19		10	32		15	27	
Lymph nodes			0.030			0.025			0.594
Negative	50	42		39	16		34	58	
Positive	46	18		53	48		21	43	
ER status			0.521			0.503			0.545
Negative	20	10		9	21		12	18	
Positive	76	50		46	80		43	83	
PR status			0.233			0.513			0.409
Negative	25	21		18	28		20	26	
Positive	71	39		37	73		33	75	
HER2 status			0.074			0.371			0.066
Negative	78	55		45	88		43	90	
Positive	18	5		10	13		12	11	

## Data Availability

The data used to support the findings of this study are available from the corresponding author upon request.

## References

[1] van Schooneveld E., Wildiers H., Vergote I., Vermeulen P. B., Dirix L. Y., Van Laere S. J. (2015). Dysregulation of microRNAs in breast cancer and their potential role as prognostic and predictive biomarkers in patient management. *Breast Cancer Research*.

[2] Siegel R. L., Miller K. D., Jemal A. (2015). Cancer statistics, 2015. *CA: a Cancer Journal for Clinicians*.

[3] Hassan M. S., Ansari J., Spooner D., Hussain S. A. (2010). Chemotherapy for breast cancer (review). *Oncology Reports*.

[4] Longley D. B., Johnston P. G. (2005). Molecular mechanisms of drug resistance. *The Journal of Pathology*.

[5] Lagos-Quintana M., Rauhut R., Lendeckel W., Tuschl T. (2001). Identification of novel genes coding for small expressed RNAs. *Science*.

[6] Bartel D. P. (2004). MicroRNAs: genomics, biogenesis, mechanism, and function. *Cell*.

[7] Ebert M. S., Sharp P. A. (2012). Roles for microRNAs in conferring robustness to biological processes. *Cell*.

[8] Geretto M., Pulliero A., Rosano C., Zhabayeva D., Bersimbaev R., Izzotti A. (2017). Resistance to cancer chemotherapeutic drugs is determined by pivotal microRNA regulators. *American Journal of Cancer Research*.

[9] Wang W., Zhang L., Wang Y. (2017). Involvement of miR-451 in resistance to paclitaxel by regulating YWHAZ in breast cancer. *Cell Death & Disease*.

[10] Liang Z., Feng Q., Xu L., Li S., Zhou L. (2017). CREPT regulated by miR-138 promotes breast cancer progression. *Biochemical and Biophysical Research Communications*.

[11] Xie F., Hosany S., Zhong S. (2017). MicroRNA-193a inhibits breast cancer proliferation and metastasis by downregulating WT1. *PLoS One*.

[12] Su J. L., Chen P. B., Chen Y. H. (2010). Downregulation of microRNA miR-520h by E1A contributes to anticancer activity. *Cancer Research*.

[13] Yuan L., Lv Y., Li H. (2015). Deubiquitylase OTUD3 regulates PTEN stability and suppresses tumorigenesis. *Nature Cell Biology*.

[14] Yang L., Li N., Wang H., Jia X., Wang X., Luo J. (2012). Altered microRNA expression in cisplatin-resistant ovarian cancer cells and upregulation of miR-130a associated with MDR1/P-glycoprotein-mediated drug resistance. *Oncology Reports*.

[15] Xu H., Sun J., Shi C. (2015). miR-29s inhibit the malignant behavior of U87MG glioblastoma cell line by targeting DNMT3A and 3B. *Neuroscience Letters*.

[16] Sarkar F. H., Li Y., Wang Z., Kong D., Ali S. (2010). Implication of microRNAs in drug resistance for designing novel cancer therapy. *Drug Resistance Updates*.

[17] Zhang B., Zhao R., He Y. (2016). MicroRNA 100 sensitizes luminal A breast cancer cells to paclitaxel treatment in part by targeting mTOR. *Oncotarget*.

[18] Gao J., Li L., Wu M. (2013). MiR-26a inhibits proliferation and migration of breast cancer through repression of MCL-1. *PLoS One*.

[19] Liu X., Tang H., Chen J. (2015). MicroRNA-101 inhibits cell progression and increases paclitaxel sensitivity by suppressing MCL-1 expression in human triple-negative breast cancer. *Oncotarget*.

[20] Zhang J., Liu W., Shen F. (2018). The activation of microRNA-520h-associated TGF-*β*1/c-Myb/Smad7 axis promotes epithelial ovarian cancer progression. *Cell Death & Disease*.

[21] Wang F., Xue X., Wei J. (2010). hsa-miR-520h downregulates ABCG2 in pancreatic cancer cells to inhibit migration, invasion, and side populations. *British Journal of Cancer*.

[22] Yuan X., Ma R., Yang S. (2019). miR-520g and miR-520h overcome bortezomib resistance in multiple myeloma via suppressing APE1. *Cell Cycle*.

[23] Shen Q., Yao Q., Sun J. (2014). Downregulation of histone deacetylase 1 by microRNA-520h contributes to the chemotherapeutic effect of doxorubicin. *FEBS Letters*.

[24] Su C. M., Wang M. Y., Hong C. C. (2016). miR-520h is crucial for DAPK2 regulation and breast cancer progression. *Oncogene*.

[25] Lee Y. R., Chen M., Pandolfi P. P. (2018). The functions and regulation of the PTEN tumour suppressor: new modes and prospects. *Nature Reviews. Molecular Cell Biology*.

[26] Marcotte R., Sayad A., Brown K. R. (2016). Functional genomic landscape of human breast cancer drivers, vulnerabilities, and resistance. *Cell*.

[27] Song M. S., Salmena L., Pandolfi P. P. (2012). The functions and regulation of the PTEN tumour suppressor. *Nature Reviews. Molecular Cell Biology*.

